# Exploration of a Swedish community-based family-oriented setting for promoting healthy food habits: professionals’ experiences

**DOI:** 10.1093/heapro/daac030

**Published:** 2022-04-01

**Authors:** Isabelle Mulkerrins, Lena Gripeteg, Christina Berg

**Affiliations:** Department of Food and Nutrition, and Sport Science, University of Gothenburg, Box 100, 405 30 Gothenburg, Sweden

**Keywords:** healthy eating, health literacy, food literacy, settings approach, family

## Abstract

Health is created in everyday life and settings, where food literacy (FL) and health literacy (HL) are recognized as important for autonomy over one’s own health. Though it is acknowledged that bridging the gap between healthcare settings and community settings may be necessary to reach those requiring extra support. Open preschool (OP) is a low threshold community setting which parents with their young children can visit voluntarily, where the various activities offered can be opportunities to address topics related to food and health. The aim of this study is to explore preschool teachers and health practitioners’ thoughts and experience of OP as a setting for health promotion, with focus on food and eating. The study is localized to OP’s in a multicultural district in Gothenburg, Sweden. Semi-structured interviews were conducted with three preschool teachers and seven health practitioners (one dental hygienist, three dietitians and three public health strategists). With an inductive approach, data were analysed using qualitative content analysis. From the perspective of teachers and health practitioners, the findings indicate that OP can be a setting for mutual learning in a supportive environment. OP provides various possibilities for improved FL and HL by linking health practitioners with families, providing the opportunity to socialize and by eating together. The results also reveal that language barriers can sometimes hinder communication, and that OP does not reach all families. Continuous collaboration between OP and health organizations and practitioners along with regular use of intercultural mediators is recommended by the professionals.

## INTRODUCTION

Food habits and eating behaviours are complex phenomena. Nutrition counselling in health care and written information are important tools in promoting healthy diets but efforts need to go beyond that to support families in acquiring the knowledge and skills to make food choices and create eating patterns linked to well-being and health.

There is evidence that public health strategies using community engagement have effects on health outcomes, health behaviours, empowerment, health literacy (HL), self-efficacy and perceived social support. Positive impacts include improving healthy eating and reducing obesity (Cyril *et al.*, 2015; O'mara-Eves *et al.*, 2015). Effects on policy and practice change resulting in enhanced sustainability and equity are also documented (Wallerstein and Duran, 2010). In the same way as gyms, sport clubs and community halls are used to promote physical activity, settings and engaging communities are needed for promoting healthy food habits. Contexts where people communicate food-related issues and ideally also make food choice decisions and perform food behaviours such as selecting, preparing and eating food.

### Food and health literacy

HL is defined as having the competence and skills to acquire, understand, evaluate and use information to maintain and promote one’s own, and one’s family’s health (Sorensen *et al.*, 2012; World Health Organization, 2013). Similarly, food literacy (FL) comprises the competence and skills to select, plan, manage, prepare and eat food to meet needs and wishes in the household (Vidgen and Gallegos, 2014; Truman *et al.*, 2017). HL and FL can be considered as key components for an individual to improve or maintain their health and make healthy food choices. However, HL and FL are complex, multifaceted and situational-based concepts, meaning individuals can be food and health literate in one context, but not in another. When major life course changes occur, such as becoming a parent or moving to a new country, food choice values, contexts and actions may also change (Sobal and Bisogni, 2009). A new parent may feel unsure about what is best for their child when it comes to food, meals and breastfeeding and therefore want information and support (Velardo and Drummond, 2013; Tartaglia *et al.*, 2021). Likewise, individuals who have migrated may face difficulties when navigating to a new country, including orientating themselves in the new society, learning a new language, and adapting to new foods and food stores, which in turn can challenge their HL and FL, and subsequently their health and well-being (Berkman *et al.*, 2011; Terragni *et al.*, 2014; Wangdahl *et al.*, 2014, 2018; Gele *et al.*, 2016; Martensson *et al.*, 2020).

Among adults, increased HL has been described as the result of providing information and structured education along with using effective communication (Nutbeam *et al.*, 2018). Encouraging integration and facilitating the utilization and development of skills is proposed vital for improved HL (Nutbeam, 2008). The importance of health literate organizations and professional’s for meeting the needs of migrants has also been explained (Wangdahl *et al.*, 2014; Martensson *et al.*, 2020). Likewise, collaboration among stakeholders is recognized beneficial (Riza *et al.*, 2020). This corroborates with the World Health Organization’s (World Health Organization, 2013) statement that ‘initiative to build health literacy must be grounded in the settings of everyday life’. Correspondingly, improved FL among parents has been reported as the result of providing nutrition education and cooking programmes in community settings (Begley *et al.*, 2019; Garcia *et al.*, 2020). Similarly, social interactions, education and support for parents (Tartaglia *et al.*, 2021). Practical activities that provide opportunities to practise food and cooking behaviours or try new tastes has further been depicted advantageous for improved FL among parents with young children (Hollywood *et al.*, 2018).

### OP as a setting for promoting health

OP is a place where parents can meet others to socialize and where children can play together. It does not cost, or require a pre-registration and families visit voluntarily how often and when suitable based on their own premises. The visiting hours for OP vary, though it is common that they are open 20 h a week where families can drop-in during the day. Unlike regular preschool, OP prerequisites that at least one parent is with the child (age group 0–6 years) the whole time. Different activities that support the parent and child’s learning and development are provided (Skolverket, 2000). Given that OP does not fall under the jurisdiction of the preschool curriculum, it is up to each OP to determine which activities they offer (Skolverket, 2000, 2019). Though this can be considered a strength of the setting due to the possibility to listen to the needs of the visiting families and provide activities and resources which are relevant and requested. Which thus, encourages involvement and participation (Törner, 2018).

OP was established in Nordic countries with the intention to minimize social inequalities, and to promote health among families with young children (Skolverket, 2000). It has been described as a low threshold setting in the local community and many are financed by municipalities (Kaiser *et al.*, 2020). In 2020, there were 495 OPs throughout Sweden, whereof half of them are part of family centres, which entails that there is a childcare centre, midwives and social services in the same building.

The aim of this study is to explore preschool teachers’ and health practitioners’ thoughts and experiences of OP as a setting for health promotion, with focus on food and eating.

The research questions guiding this study are what do personnel in preschool, public healthcare and public dental care mention about (i) aspects of OP as a setting for health promotion, where parents, children, teachers, and health practitioners can improve their FL and HL? and (ii) aspects of food and eating related to health and well-being addressed at OP?

## METHODS

### Study setting

This qualitative interview study was carried out during September 2020–February 2021 and was localized to the district Angered in the North-East of Gothenburg, Sweden. Angered is a district in the suburbs of Gothenburg where many have low income and formal education, and an estimated 50% of the inhabitants are foreign born. It has been reported that 61% of adults living in Angered reported their health as good compared to 82% of adults living in a district which is situated central in Gothenburg City (Lundquist, 2017). This is congruent with objective measurements which have reported higher rates than average of lifestyle related illnesses among inhabitants living in North-East Gothenburg in comparison to other districts in Gothenburg (Olsson and Panfilova, 2009).

In this district, collaborations between healthcare professionals/organizations and OP are currently occurring.

According to public statistics (Gothenburg City, 2021), 57% of families with children 0–6 years have visited OPs in Gothenburg while 18% do not know what OP is. More than a quarter visited weekly. Parents with tertiary education, high income and those who were born in Sweden were more likely to visit OP than other groups. However, one-third of families who had migrated to Sweden during the last 5 years had visited. It should be noted that the data available is from 2020–2021, during the Covid-19 pandemic (the pandemic was the most common reason for not attending OP), and less than half of the invited parents took part in the survey initiated by the city of Gothenburg.

### Selection procedure and participants

Purposive sampling was used to recruit individuals. All six OP in the district were invited by e-mail to be interviewed. When followed up by phone calls, five OP showed interest. Two of them were not able to partake due to the Covid-19 pandemic. The reason was that all OPs were closed and the personnel was relocated to ordinary preschools with uncertainty of the future. The three OP who agreed to participate, selected one preschool teacher each to be interviewed.

To also explore the perspective of health practitioners, dietitians and other professionals performing health education related to food and eating at OP were relevant to contact as well as public health strategists responsible for public health activities in Angered. Three public health strategists from public health units at health care and dental health were invited to be interviewed and all agreed. In turn, they suggested two dietitians and one dental hygienist as potential participants. These health practitioners and one further dietitian (suggested by one of the preschool teachers) were contacted and agreed to participate. During the interviews, other suggestions for health practitioners to interview were asked for, but no additional names were mentioned.

Ten individuals were interviewed: three preschool teachers from different OPs which are part of family centres in Angered and seven health practitioners whereof three dietitians, one dental hygienist and three public health strategists working in different public healthcare organizations. All participants were female, and their time worked at or in collaboration with OP varied from several months to years.

### Data collection and ethical considerations

Qualitative semi-structured interviews were utilized to help explore and understand the participants’ experiences and thoughts. The interview guide varied to some extent for the health practitioners and preschool teachers due to different experiences of OP. General themes explored were their thoughts and experience of OP as a setting for promoting healthy food habits, what they have learnt from working at OP and what support/information they perceived families wanted. Follow-up questions were asked in accordance with the direction of the interview with the intention to gather relevant insights.

Nine interviews were conducted online via Zoom, and one was done face to face at the individual’s workplace. The interviews lasted between 40 and 75 min. All interviews were conducted in Swedish by the first author, recorded and transcribed verbatim.

The possibility for a power imbalance during interviews with the preschool teachers was recognized due to the role of the interviewer and their area of expertise, which could elicit social desirability in answers. To minimize this risk, open questions with the intent to explore thoughts and experiences were asked along with refraining from questions which could be perceived judgemental.

Prior to agreeing to the interview, all participants received an information letter informing them about the objective of the study, that their contribution was done voluntarily, that the interview would be recorded and data would only be accessed by internal sources. Either oral or written consent was provided before the interviews. The ethical principles were reiterated at the start of each interview whereupon participants also had the opportunity to ask questions. Due to the specificity of individuals’ who could provide relevant insights, it could compromise their anonymity. When finalizing the manuscript all interviewees gave their written consent concerning the description of participants and quotations used.

### Data analysis and presentation of results

Data analysis began after seven interviews were transcribed and therefore occurred simultaneously as three more interviews were done. Similarities in content between the interviews being conducted and those being analysed were observed.

An inductive approach to analyse data was implemented. Following the methodological approach by Graneheim and Lundman (Graneheim and Lundman, 2004), qualitative content analysis to emanate both the manifest and latent meaning was used. Two separate analyses of data were done, one for each research question.

Before the analysis began, the interviews were read in their entirety several times for a comprehensive understanding of the data. Subsequently, meaning units that provided answers to the research question were selected and coded from each interview. The codes were created with the intent to reflect the manifest meaning, thereby many codes used the same words as those from the meaning unit (Graneheim and Lundman, 2004). Coding was repeated for all the selected meaning units. By contrasting and comparing codes, those with similar content were grouped together and subcategories began to form. As suggested by Graneheim *et al.* (Graneheim *et al.*, 2004), the subcategories were created to be heterogeneously exclusive and differentiate from each other to show nuances of the data and category description. The main category names reflect a higher abstraction of data from the associated subcategories (Graneheim *et al.*, 2017). The final step included an abstraction of data where the latent meaning was elucidated in one overarching theme for the analysis of the first research question. The analysis related to the second research question followed the aforementioned steps however focus was solely on the manifest meaning and no higher abstraction of data was done.

Throughout both analyses, a continuous back-reference to the entire interviews along with the aim and research question(s) was done to ensure a valid and representative analysis in accordance with the manifest meaning. Three authors, with expertise in nutrition and health promotion, and differing insight into the research topic partook in the data analysis. This contributed to variation in perspectives and minimized the risk for internal bias of the analysis. The coding was done by the first author, although discussion and reflection were recurrently done throughout the analysis until agreement between all three was achieved.

The participants were contacted and given the opportunity to provide feedback on the results. The consensus of response given was positive and stating that they had nothing further to add. Additionally, at a meeting with corresponding healthcare professionals and preschool teachers, the study findings were presented whereby they were asked to provide feedback on the results. The response given signified that the results coincide with that of their own experience of OP.

The quotations selected are intended to support, illustrate and confirm the presented results. Quotations were translated from Swedish to English at the final stage of the analysis by the first author who is bilingual.

## RESULTS

### OP provides a meeting place for mutual learning in a supportive environment

**Fig. 1:**
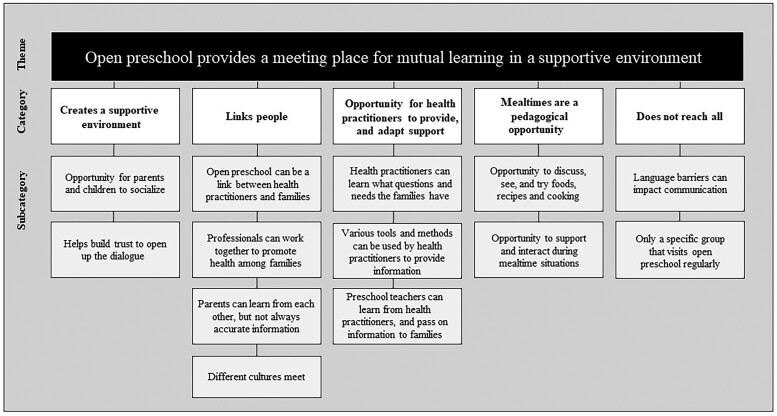
An overview of the subcategories, categories and theme concerning aspects of open preschool as a setting for health promotion where food and health literacy can be improved.

The interviewees describe that parents’ often have many questions and queries related to food and health. Various opportunities for improving FL and HL were mentioned with the underlying understanding that they are made possible by OP providing a meeting place for mutual learning in a supportive environment. A place where parents, children, preschool teacher and health practitioners such as dietitians and dental hygienists *can* learn from each other. An overview of the categories which resulted in this overarching theme are presented in Figure 1. The five categories and subcategories are described accordingly.

An important characteristic of OP was described as the ability to create a supportive and welcoming environment where the families should feel involved. It was further expressed that OP is a setting where different cultures and individuals (parents, children, teachers, and different health practitioners) meet. One of many activities are eating, tasting and preparing foods together, and thus conversations about food and eating can occur naturally. This is also an opportunity for preschool teachers to address the everyday situations and questions parents have related to food and health while the health practitioners can contribute with support and information in their field of expertise. Health practitioners were described to visit OP on request by both parents and preschool teachers. The collaboration also entailed regular meetings, for example, the dental hygienist met families at one OP weekly and the dietitians visited all OP several times a year.

They [OP] have a very large role. Especially the everyday situation, what happens every day. That they are a meeting place for it. (Interview 2, Dietitian)

What a fantastic setting to meet families in. And so exciting when the parents accompany their children. Preschool is one thing, but there you do not meet the parents, but here they have time, and you can talk to them. (Interview 9, Public health strategist)

### Creates a supportive environment

OP was described as a setting for children to play and learn while still in the safety of their parents. It was expressed that some parents with young children, especially those who are new to the country, can feel lonely and may long for a social context, which OP can provide. It was also mentioned that social interactions at OP provide a chance for families to practise and receive support with the Swedish language.

The teachers mentioned that trust can be built between themselves and the families, which was viewed as highly important. The teachers describe being able to notice when the parents are susceptible to having conversations about food and health, and subsequently can start the conversation in informal ways. Building trust to one another along with the supportive environment was alluded to also facilitating families in asking questions or daring to ask for help.

### Links people

It was indicated that OP can link families with health practitioners. The participants describe that some families are new in the country and may not know where or how to seek healthcare. Whereby the health practitioners also mentioned that some families may get their first contact with a dietitian and dentist at OP, where the setting can be perceived as less intimidating. This was described as making it easier for the families to seek help—and know where to seek help—in the future. The public health strategists endorsed continuous collaboration with settings such as OP.

You must make an effort to reach those groups who do not spontaneously seek medical care themselves, or health care. Because it is generally those who have the worst health…so we work together with professionals in the local community such as those who work at open preschool for example. (Interview 1, Public health strategist)

We need to meet people in their local areas, where they live naturally. And we need to do it in a simple, easily accessible way. (Interview 8, Public health strategist)

Upon request from families, or during scheduled visits, different professionals can meet and cooperate at OP, thereby providing information and support in numerous areas such as nutrition, oral health and physical activity. This also contributes to a family-centred approach, which was recurrently alluded to, meaning that both parents and children are supported in different ways.

Questions and concerns regarding food and health can be voiced by the families. Though noteworthy is that all the interviewed preschool teachers reiterate that their area of expertise is not food or health and therefore the collaboration helps them refer parents to experts when necessary. They described their primary role in the collaboration as contributing with the pedagogical role and providing support, as exemplified in the following quote:

It is very important that we do not go in or take upon ourselves something that is not actually within our competence. So, I think our competence is to see that here we can offer support. (Interview 4, Preschool teacher)

The participants also mentioned that parents can share advice, tips and recipes with each other at OP; however, they also noted that the information shared between parents is not always accurate, thus having experts to provide correct information or moderate the discussion was described beneficial.

As Angered is a multicultural district, OP in this area was described as a setting where different cultures meet, and mutual learning of each other’s food traditions and culture can occur. OP was depicted as a setting where migrants can learn how to navigate in the new country. In addition, the health practitioners highlighted the importance of being humble and open for the differences in food culture, and further portrayed the meetings with families as educative for themselves.

Then you understood that oh, here are women from all over the world … who have completely different food cultures and preferences and stores that they shop in. That it is difficult to go there with a uniform message that suits everyone. (Interview 10, Dietitian)

### Opportunity for health practitioners to provide and adapt support

Meeting families at OP is a chance for the health practitioners to learn and understand what type of information, and support, they need or want. Although, not all the health practitioners explicitly described that it was a possibility for bidirectional learning when meeting families. There was possibility for health practitioners to answer questions both in group settings and individually allows for parents and teachers to listen and learn even if not actively participating or asking questions.

All the health practitioners implied that OP provides a setting where a conversation can be encouraged and thereby information can be provided based on the questions posed by the families. However, it was noted that parents do not always directly ask questions. Although, different methods and tools could be used to instigate a dialogue and communicate about food and health. Examples mentioned included pictures, discussion cards, video material as well as use of visual displays. OP also provides opportunities for practical activities with food where concrete tips can be given. The need to use visual tools is reiterated by the teachers, as seen in the following quote.

Because in fact, not everyone can read. And some can read, but maybe not in Swedish. (…). But then a picture says more than all those words. (Interview 3, Preschool teacher)

The teacher’s role was expressed as important, especially as they can see, address and support the families with everyday situations regarding food, eating and health, and they can discuss those topics frequently. That teachers also learn from health practitioners is considered beneficial, as exemplified in the following quotation.

That the dietitian comes to the open preschool is good both for the parents, but also for the teachers. So, they can sit and listen and hear (…) and they can do the same later if the parents have similar questions when the dietitian is not there. (Interview 7, Dietitian)

The teachers corroborate this, by mentioning that they can learn from the health practitioners and in turn support families. It was also described as positive when both the teachers and health practitioners gave the same advice as it could otherwise be confusing for the families.

### Mealtimes are a pedagogical opportunity

Meals were often eaten together and primarily initiated by preschool teachers but also by families or health practitioners. The preschool teachers described that OP provides food items for snacks, breakfast or festivity, and parents sometimes bring foods for their child or to share. Mealtimes at OP were described as providing a significant opportunity to promote healthy eating. Healthy foods and snacks could be shown, offered and tried by the families, subsequently dialogue about food could occur naturally. Although it was not a frequent occurrence, there was mention of opportunities to cook together, where practical cooking skills, adapting recipes and showing kitchen appliances could be demonstrated or practised. The health practitioners encouraged OP’s to continue with different practical activities related to cooking, food and eating.

Moreover, mealtimes were seen as pedagogical for both children and parents due to the interactions and support which could occur, as illustrated by the following quote:

(…) that the children and parents get to see other children in similar ages who introduce food and that can help you challenge a little more and get more ideas and inspiration. (Interview 2, Dietitian)

It was further expressed that the teachers can help create calm mealtimes and limit distractions for the children while eating. This in turn supports the parent(s) and helps set an example of a good mealtime situation.

#### Does not reach all

The category ‘does not reach all’ indicates that OP may not always be a setting for improved FL and HL, particularly if certain groups cannot be reached. The interviewees acknowledged that it was primarily mothers with their young children, who visited OP regularly. It was expressed that reaching fathers or other family members would be beneficial. Families who have migrated to Sweden were described to visit OP. Similarly, the interviewees recognized that those who visit OP frequently are the ones who have received information about what OP is and have an interest in receiving support or wanting to socialize. Thereby missing families who may need extra support, but not know where or how to get it. Therefore, the difficulty was seen in reaching those that do not visit OP regularly, or at all.

Both teachers and health practitioners noted that language barriers could hinder communication. Though, it was described that they find ways around the language barrier, including use of different communication tools as previously mentioned, using online translators, or that other families could help translate. But if the information was fully understood, was not always known. Use of interpreters was not a frequent occurrence, though regular use of authorized interpreters and intercultural mediators was recommended to facilitate communication.

### Aspects of food and eating related to health and well-being addressed

Numerous topics related to promoting health can be addressed at OP. Seven different aspects of food and eating related to well-being and health that were recurrently highlighted are presented in Table 1. Example, showing and informing about healthy food choices and how to adapt recipes. Supporting parents with breastfeeding and food introduction for children as well as supporting them in their parent role. Limiting sugar was repeatedly described from various perspectives, whereby it was mentioned that information about sugar and how/why it should be limited needed to be addressed frequently and more predominantly.

**Table 1:** Aspects of food, and eating, related to well-being and health commonly addressed at open preschool according to the interviewed preschool teachers and health practitioners

Categories of addressed aspects of food and eating at open preschool	Description of each category	Illustrating quote
Healthy food choice and nutrients	Healthy eating for both child and parent(s) according to the ‘keyhole symbol’, ‘plate model’, ‘food circle’ and the Swedish national nutrition recommendations Suggestions for healthy food choices and eating varied Nutrition advice about vegetarian diets, organic food and allergies Information about vitamins and minerals such as iron and vitamin D Myths and claims from the media, e.g. FAD^a^ diets and heavy metals in baby food	‘Partly, as I said, if the family had specific wishes. It can be anything from questions about salt and sugar to snacks and vitamins and so on’ (Interview 7, Dietitian)
Limiting sugar	Limiting sugary foods at open preschool Talking about why/how to limit sugar for children Health and dental consequences of too much sugar Looking at ingredients and nutritional value (sugar content) of popular baby products Visual display of sugar in snacks, and sugar pamphlets available	‘We have a ‘hidden sugar’ pamphlet, and we have made a sugar cupboard where we show how much sugar there is in each…in different ordinary food items’ (Interview 5, Dental hygienist)
How to make and adapt healthy meals and snacks	Cooking and preparing baby food portions and healthy snacks, e.g. fruit puree, banana-egg pancakes, sandwiches and hummus Adapting recipes and showing different kitchen appliances to use when preparing food Recipe handouts	‘Then they [dietitians] have come and shown, example, how to make porridge sticks. There are many who make porridge and then there is porridge leftover. And what do you make of it? Or the children do not like it […] and then you make a variation, and they still get the nutrients. And they have made smoothies and brought with them pamphlets that the parents have been given and they have gotten recipes…’ (Interview 3, Preschool teacher)
Breast feeding and food introduction for children	Breastfeeding routines; when to stop breastfeeding, breast feeding during the night, bottle feeding Courses such as the ‘breastfeeding café’, ‘food in pieces’, ‘taste for food’ Food introduction, including baby-led-weaning Children can try different consistencies and portions	‘Yes, it is everything from when and what the baby can start eating, ‘do I dare give this?’ It starts during breastfeeding you can say.’ (Interview 4, Preschool teacher)
Parent and family well-being	Setting boundaries for children Creating calm mealtime situations Parenting education courses addressing child development and bonding Parental well-being and struggles of being a parent	‘And then it is not just about eating habits, but also parental support and responding to children's reactions and their own feelings and so on. And that in itself affects the diet or the diet in general’ (Interview 2, Dietitian)
Navigating in a new country	Increasing trust in the healthcare system Cultural food habits and the nutritional impacts of different traditions Various ways to eat food(s), e.g. vegetables on the side or *in* the dish Foods being controlled and safe to eat (due to religious reasons) Different food cultures and traditions, e.g. Swedish midsummer and Christmas celebrations	‘Knowledge about Swedish food, that it can be safe and secure that you understand the list of ingredients, and that the control over ingredients in food sold in Sweden is controlled and that it is safe and such things’ (Interview 2, Dietitian)
Contact with health practitioners, where and how to seek help related to food and eating	Dietitian Dental hygienist Midwife Nurse	‘If you talk about food and eating habits and so on, then you can [say] ‘you can call the dietitian here’ and you can give suggestions and do the same with the public dental service.’ (Interview 6, Preschool teacher)

a A FAD diet is a restrictive diet that quickly becomes popular via media, often promising quick weight loss or improved health by excluding certain foods or entire food groups.

The variation in topics is possible due to collaborations both within the family centres (preschool teachers, midwives and nurses) and with professionals from public healthcare organization such as dietitians, dentists and dental hygienists.

## DISCUSSION

The presented findings indicate how OP enables activities which can improve knowledge and skills to acquire, understand, evaluate and communicate information about food and healthy eating, which are associated with improved FL and HL (Sorensen *et al.*, 2012; Vidgen and Gallegos, 2014; Truman *et al.*, 2017). Subsequently, these activities may promote healthy food and eating habits in parents and their children.

### Activities and opportunities which enable improved FL and HL

It was described that at OP, frequent dialogue concerning aspects of food, eating and health could occur naturally. This makes it possible for parents, teachers and health practitioners to communicate about food and eating, which thereby can contribute to mutual learning. The results further portray that the possibility to build trust to one another is a valuable characteristic of the setting, as it could decrease the threshold among families when asking for help, while also making parents more susceptible to have conversations about food and eating, which can be considered sensitive topics. Similar results have previously been reported from the perspective of parents visiting OP (Fryckholm, 2020; Alm *et al.*, 2021). As it was expressed that parents were encouraged to ask questions and partake in activities, it enables parents to acquire, understand, apply and to some extent critically appraise information they receive. Sorensen et al. (Sorensen *et al.*, 2012) has explained that these competencies are crucial for HL, while Nutbeam (Nutbeam, 2000) has similarly suggested that participation and integration are integral for improved HL and autonomy over one’s own health. Additionally, linking families with health practitioners may facilitate families in gaining the knowledge and understanding of how to navigate the healthcare system, such as where and how to seek help.

It was depicted that numerous food-related activities can occur at OP which enables families to gain knowledge and skills required to take decisions and make desired food choices. Activities included eating together, cooking and baking which facilitates families in obtaining, understanding and applying skills and knowledge related to selecting, preparing, cooking and adapting foods and meals. Particularly, in relation to food introduction for babies and making healthy meal for the child. Likewise, it allows both children and parents to test, and appraise different, and possibly new foods.

The previously reported importance of mealtimes at OPs (Adolfsen *et al.*, 2012; Kaiser *et al.*, 2020) was also highlighted by the participants in the present study. They described that during meals, conversations concerning food and eating could occur and is therefore an opportunity for families to share tips and advice while also receiving support and information from professionals. An example of this is related to limiting sugar, as it was described that preschool teachers along with parents could examine the ingredients of baby food to see the sugar content, and then discuss why sugar should be limited. Activities such as this, along with health practitioners being able to debunk false nutrition claims from media may contribute to a critical knowledge acquisition concerning food choices and health. Eating together further presents the opportunity for the children to get inspired and learn from one another.

These activities and opportunities are important as there are few settings for parents with young children where so many different aspects of food and eating can be addressed, including access to information, support and practical activities. Settings such as food stores or within health and dental care may address various aspects of food and eating but not with as much diversity. Also, there may not be the possibility to use different methods and tools to communicate, and adapt information in those settings, as it was expressed can be done at OP. Although, it can be considered that the combination of a supportive setting, where different individuals can meet, dialogue can occur, and activities are provided is what contributes to the possibility for OP to enable families, teachers and health practitioners to cultivate various skills and knowledge required to improve HL and FL.

### The significance of OP for migrants

Previous studies have revealed that health information does not always reach migrants, and is explained by factors such as language barriers, mistargeted information or information not being readily available to this group (Gele *et al.*, 2016; Martensson *et al.*, 2020). As an estimated 50% of inhabitants are migrants in Angered, it stresses the importance of understanding how a community-based setting may support those who need extra support and information related to healthy eating.

Studies both in Scandinavia and internationally have reported that migrants may have their HL and FL challenged when in a new country. Even if migrants are a very heterogeneous group, some general explanations for this have been identified. It is partially explained by difficulties navigating in the new country, not knowing where or how to seek health and medical care due to lack of readily available information, and not being able to access or understand health information (Wangdahl *et al.*, 2014, 2018; Gele *et al.*, 2016; Martensson *et al.*, 2020; Ringsberg *et al.*, 2020). Correspondingly, aspects which can challenge migrants FL have been described as language barriers, cultural differences, mistrust in ingredients and food labels, fear of certain foods with respect to one’s religious beliefs and uncertainty of how to adapt traditional recipes based on local foods available (Garnweidner *et al.*, 2012; Terragni *et al.*, 2014; Popovic-Lipovac and Strasser, 2015). Whereby these can contribute to less healthy food choices and may impair well-being by making preferred food choices difficult (Mellin-Olsen and Wandel, 2005; Wandel *et al.*, 2008). Language barriers which could impact understanding of information was acknowledged by both the health practitioners and teachers, therefore an established co-operation with intercultural mediators when health practitioners visit is recommended to assist communication.

As OP is intended to provide support and activities based on the needs of the visiting families, it provides ample opportunity to help overcome some of those barriers, including the chance to supply relevant information that is understandable, along with providing practical activities and concrete tips. However, it is also recognized that the collaboration with different health practitioners, including those part of the family-centre helps contribute to the wide range of topics addressed.

The findings of this study highlight that OP can provide a setting where many of the aspects challenging migrants HL and FL can be addressed. This meeting place enables targeted activities according to the visitors’ requests, wishes and needs. However, the interviewed teachers and health practitioners also reflected upon the difficulty in reaching those who do not know what OP is or what it can contribute with. The interviewees also expressed that families who visit OP may be those already integrated into the Swedish society and have an interest in receiving support. This meaning, that the challenge is to increase awareness of what OP is, and how it can help, along with encouraging participation for all families with young children in the community.

Previous studies have suggested that community engagements which bridge the gap between public healthcare settings and community settings may be an advantageous strategy for health practitioners, as to reach and support certain groups with their challenges in making desired healthy food choices (O'mara-Eves *et al.*, 2015; Bulling, 2017; Bulling and Berg, 2018; Ringsberg *et al.*, 2018; Riza *et al.*, 2020; Alm *et al.*, 2021). The present study shows that OP has a beneficial role as it enables different professionals to collaborate and work together.

### Strengths and limitations

A strength of this interview study includes contributing to the limited research concerning how a community-based family-oriented setting can promote healthy food habits. The variation in interviewees, both in occupation and experience, provided diversity in perspectives and is therefore a strength of the study. On the other hand, a limitation is that not all OP’s in the district are represented by teachers. At the time of the study, all OPs in the district had to close due to the Covid-19 pandemic, and several preschool teachers who had been contacted and shown interest, had to decline participation due to the restrictions. Furthermore, a risk that participants portray positive biased views cannot be disregarded. However, the interviews explored both positive and negative aspects of OP as a setting for promoting healthy food habits. The health practitioners do not only collaborate with OP but work in different settings with different strategies. Likewise, the teachers focus on many activities not related to food and eating in OP. As the results are from the perspective of the health practitioners and OP teachers, it cannot be said whether HL and FL are improved among families. Neither do it describe their views about OP as a setting for health promotion. Exploring parents’ thoughts and experiences are therefore planned when the Covid-19 restrictions are lifted.

## CONCLUSION

This study concludes how settings, such as OP, can have a role in promoting healthy food habits. The presented findings highlight the various possibilities at OP which can facilitate improved HL and FL with the understanding that creating a supportive environment, and linking individuals is paramount in doing so. It also allows health practitioners and teachers to understand what information and support families need. Together, these can help families in increasing FL and HL. However, OP may not reach all relevant families. Therefore, it is recommended to increase awareness of OP in the community setting as well as work with intercultural mediators. Finally, it is recognized that active engagement from all stakeholders is likely a necessity for improved FL and HL at OP.
